# Vegetation and Cold Trapping Modulating Elevation-dependent Distribution of Trace Metals in Soils of a High Mountain in Eastern Tibetan Plateau

**DOI:** 10.1038/srep24081

**Published:** 2016-04-07

**Authors:** Haijian Bing, Yanhong Wu, Jun Zhou, Rui Li, Ji Luo, Dong Yu

**Affiliations:** 1 Alpine Ecosystem Observation and Experiment Station of Gongga Mountain, The Key Laboratory of Mountain Surface Processes and Ecological Regulation, Institute of Mountain Hazards and Environment, Chinese Academy of Sciences, Chengdu 610041, China

## Abstract

Trace metals adsorbed onto fine particles can be transported long distances and ultimately deposited in Polar Regions via the cold condensation effect. This study indicated the possible sources of silver (Ag), cadmium (Cd), copper (Cu), lead (Pb), antimony (Sb) and zinc (Zn) in soils on the eastern slope of Mt. Gongga, eastern Tibetan Plateau, and deciphered the effects of vegetation and mountain cold condensation on their distributions with elevation. The metal concentrations in the soils were comparable to other mountains worldwide except the remarkably high concentrations of Cd. Trace metals with high enrichment in the soils were influenced from anthropogenic contributions. Spatially, the concentrations of Cu and Zn in the surface horizons decreased from 2000 to 3700 m a.s.l., and then increased with elevation, whereas other metals were notably enriched in the mid-elevation area (approximately 3000 m a.s.l.). After normalization for soil organic carbon, high concentrations of Cd, Pb, Sb and Zn were observed above the timberline. Our results indicated the importance of vegetation in trace metal accumulation in an alpine ecosystem and highlighted the mountain cold trapping effect on trace metal deposition sourced from long-range atmospheric transport.

Mountains, especially remote alpine areas, are more sensitive to global changes than surrounding lowlands. With increasing human activities, larger quantities of compounds, such as toxic trace metals, persistent organic pollutants, and sulfur and nitrogen oxides, are released into the atmosphere. Mountains have been found to preferentially accumulate these compounds carried by long-range atmospheric transport due to cold trapping effect^1^. Furthermore, mountains are more sensitive to climate changes than other ecosystems^2^. The global warming and acidic deposition most likely result in the remobilization of environmentally important compounds as ice and snow melt, thereby threatening alpine ecosystems themselves and fresh water downstream. The accumulation of elements in soil plays an important role in the safety of the alpine ecosystem. Elements, such as Cu and Zn, are essential for various metabolic processes in trace amounts in organisms, whereas they can cause adverse effects at higher concentrations. Other trace metals (*e.g.,* Ag, Cd, Pb and Sb) are toxic to organisms. Research on the distribution patterns and sources of trace elements, as well as the influence of various factors (*e.g.,* precipitation, vegetation, human activity) on their behavior in alpine ecosystems is of great importance.

In high mountain areas, the differences in elevation form a marked climatic gradient, which determines the vegetation distribution and soil development, and affects the deposition and storage of trace metals. Insights of the distribution patterns of trace metals along the elevation gradient would be valuable for the evaluation of the regional and/or local anthropogenic versus natural contributions under the effects of vegetation and mountain trapping capacity. Many researchers have examined the elevation-dependent patterns of trace elements in different mountains around the world based on fresh snow^3^,^4^, moss^5^,^6^, fern^7^, lichen^8^ and soil^9^,^10^. However, a consistent trend of trace metal accumulation with elevation has not been observed due to local or regional reasons. Additionally, there is little comparability among these studies, because the elemental accumulation in fresh snow, fern and moss represents a short-time scale process that can be influenced by many factors, such as wind and precipitation in a certain period. The elemental accumulation in soil, however, is a long-term process; thus, the distribution of trace elements in soil can be more representative of their elevation-dependent patterns than other indicators (*e.g.,* moss, fern, etc.) at longer time-scales.

Gongga Mountain (29°20′–30°20′N, 101°30′–102°15′E, 7556 m a.s.l. (above sea level)) is the highest mountain on the eastern edge of Tibetan Plateau. Mt. Gongga is an effective orographic barrier to aerosol transport due to its large elevation gradient. Natural aerosols with particles between ca. 2 and 200 μm tend to settle out of the atmosphere quickly under the effect of gravity, whereas anthropogenic aerosols, with particles smaller than 1 μm almost behaving like a gas or vapor, are more stable in the atmosphere and can be transported long distances with atmospheric circulation^11^. Thus, the trace elements, especially toxic metals, contained in aerosols are apt to deposit in the high mountain ecosystem as the result of the mountain cold condensation effect. The eastern slope of Mt. Gongga is characterized by large elevation gradient (1100–7556 m a.s.l.). With the soil development, a complete vegetation succession has been formed along this large gradient including broadleaf forests, broadleaf-coniferous forests, coniferous forests, shrub and meadow. This eastern slope is mainly influenced from the Asian monsoons from southerly and southeasterly air mass^12^. Mt. Gongga is an ideal space to investigate trace element distribution patterns in soil with elevation and to understand the dominant factors that affect this remote alpine ecosystem. In previous research, Wang *et al*.^13^ did not found clear variation of the concentrations of certain trace metals (*e.g.,* Cd, Cu, Pb and Zn) between the topsoil and subsoil layers in the sub-alpine forests on the eastern slope of Mt. Gongga and concluded a low contribution of atmospheric deposition. However, our recent research confirmed that the accumulation of Cd in the soils of the eastern slope of Mt. Gongga was mainly from atmospheric sources^14^. Otherwise, there have been few reports on the elevation patterns of trace metals in the soils on the eastern slope of Mt. Gongga.

In this work, we conducted an extensive investigation of element distributions in the soils of the eastern slope of Mt. Gongga. Thirteen elevations between 2000 and 4500 m a.s.l. were selected (Fig. 1). Along this large elevation transect, there are marked differences in precipitation and vegetation type, whereas the composition of the bedrock is generally constant (see the Methods). Therefore, we hypothesized that the elevation-dependent trace metal distribution in the soils would exhibit a close relationship to wet deposition and vegetation. Meanwhile, Southwest China is a main mine area in China including the lead-zinc ores, antimony ores, and hence the mining and smelting would release trace metals (*e.g.,* Ag, Cd, Cu, Pb, Sb and Zn) to the environment. Therefore, the objectives of this work were to investigate the elevation distribution patterns of these trace metals in the soils on the eastern slope of Mt. Gongga and to explore the dominant factors controlling their distributions. Meanwhile, other elements (Al, Ba, Ca, Co, Cr, Fe, K, Mg, Mn, Mo, Na, Ni, P, Sr, Th, Ti, Tl, U, and V) in the soils were also measured to bolster the analysis.

## Results

### The soil properties

Pedogenesis and vegetation succession resulted in the increasing soil development with the decreasing elevation. These transitions were clearly observable in the thickness of the soil profiles and the O and A horizons (Table S1). The soils were generally acidic (low pH values in the O and A horizons), and the lowest value was observed in the coniferous forest (Table 1). The concentrations of SOC decreased significantly with depth (O horizon-range: 10.7–36.6%, mean: 25.6%; A horizon-range: 4.3–28.2%, mean: 14.4%; B horizon-range: 0.5–18.5%, mean: 4.5%; C horizon-range: 0.2–7.3%, mean: 1.4%; Table S2). Although the concentrations of SOC did not exhibit notable differences in different vegetation zones, they were relatively high in the mixed and coniferous forests despite the soil horizon (Fig. S1).

### Concentrations of elements in the soils

The median concentrations of major and trace elements in the soils are presented in Table 2, as are the corresponding concentrations in the UCC^15^ and in the Chinese UCC and sedimentary layer^16^. Compared with the concentrations in the UCC, the Chinese UCC and sedimentary layer, most elements in the C horizon featured comparable values. Copper, Mo, Na and Tl had relatively lower concentrations in the C horizon, whereas Cr, P, Ti and V showed slightly higher values. Considering this difference, the element concentrations in the C horizon were regarded as their local background. The concentrations of P, Ag, Sb, Pb and Cd were higher in the O and A horizons, especially Cd, which showed significantly higher values than its local background.

The concentrations of Cu, Pb and Zn in the soils of our study were also in the range of the northeastern US-Canadian sub-alpine floor^17^, soils in the Central Pyrenees^9^,^18^, a Swiss forest^19^ and the French Alps^20^. These studies found that atmospheric deposition was the primary cause of the metal accumulation. The concentrations of Cd were largely higher in the O and A horizons of our study area than in the soils mentioned above but similar to the values reported for the northern slope of Mt. Everest, where the type of bedrock was considered as the main reason^10^. Based on the element distributions in the soil profiles (Table 2), P, Ag, Cd, Pb, Sb and even Zn in the O and A horizons were probably influenced by exogenous processes.

The distribution patterns of the trace metal concentrations in the O and A horizons along the elevation gradient are presented in Fig. 2. Two zones featured the O horizon with high concentrations of Cd and Zn (approximately 3000 and above 3700 m a.s.l.). The distribution of Ag, Pb and Sb in the O horizon changed markedly approximately 3000 m a.s.l. Copper in the O and A horizons and Zn in the A horizon decreased between 2000 and 3700 m a.s.l. and then increased with elevation.

### Enrichment factors (EFs)

Enrichment factors were used to reveal the mass balance of elements in the O, A and B horizons (Fig. 3). The EFs in the O horizon were lower than 1.5 for most elements, between 1.5 and 3.0 for Ca, Mn, Mo, Ni and Tl, and greater than 3.0 for the other elements, especially Cd which reached a median value of 79.5. In the A horizon, the EFs of Mo, P, Cu, Pb and Zn were approximately 1.5, and those of Ag, Cd and Sb exhibited slightly higher values. Otherwise, the element EFs in the A and B horizons were less than 1.5.

Similar to the concentration distributions with elevation, Ag, Cd, Pb, Sb and Zn in the O and A horizons (especially the O horizon) showed high EFs at the elevations of approximately 3000 m a.s.l., and Cd and Zn in the O horizon also enriched at above 3700 m a.s.l. (Fig. 4). The enrichment trend of Cu differed from its concentrations along the elevation gradient. A high enrichment of Cu was observed in the O and A horizons at elevations of approximately 3000 m a.s.l. and higher (Fig. 4).

### Lead isotope composition in the soils

Since the low EFs of metals in the B horizon (Fig. 4) suggested less anthropogenic contribution, the ratios of ^206^Pb/^207^Pb and ^208^Pb/^206^Pb in the O, A and C horizons were analyzed to distinguish the Pb sources in the soils (Fig. 5). The ^206^Pb/^207^Pb (mean ± SE) was significantly lower in the O horizon (1.1718 ± 0.0014), followed by the A horizon (1.1857 ± 0.0014), and the highest was observed in the C horizon (1.1956 ± 0.0017) (p < 0.05). The ^208^Pb/^206^Pb in the soils decreased in the order of O horizon (2.1043 ± 0.0013) > A horizon (2.0927 ± 0.0024) > C horizon (2.0834 ± 0.0037) (p < 0.05). Along the elevation gradient, the lower ^206^Pb/^207^Pb and higher ^208^Pb/^206^Pb in the O and A horizons were observed in both low- and high-elevation zones, and the marked change point existed at the elevation of 3614 m a.s.l.

## Discussion

### Source discrimination for trace metals in the soils

Factor analysis was applied to establish the relationships of elements in the soils, and four components with Eigenvalue higher than 1.0 were extracted (Table 3, Fig. S2). Component 1 included most elements in the soils with positive loadings for Al, Ba, Ca, Co, Cr, Fe, K, Mg, Mn, Na, Ni, Sr, Th, Ti, U, V, and negative loadings for Ag, Cd, Pb, Sb. Component 2 mainly grouped positive loadings for Co, Cu, Ni and Zn. Component 3 consisted of Cd, Mn, P, Pb, Tl, Sb and Zn with positive loadings. Component 4 exhibited positive relationship between Ag and Mo. The factor analysis distinguished the different geochemical characteristics of the elements in the soils. Although the statistical analysis could not identify the specific sources of trace metals, it suggested that the metals (*e.g.,* Ag, Cd, Pb, Sb and Zn) in the soils were influenced from non-crustal sources due to their different relationship with the lithogenic elements (*e.g.,* Al, Ca, K, Ti).

Lead isotopes have been widely used to distinguish anthropogenic Pb from natural sources^21^,^22^,^23^. Our analysis of the Pb isotope composition in the soils (Fig. 5) showed significant correlation between ^206^Pb/^207^Pb and ^208^Pb/^206^Pb (p < 0.0001), indicating that at least two kinds of sources affected the Pb distribution in the soils. Compared with the Pb isotope compositions in other materials (Fig. 5), the Pb isotope ratios in the O and A horizons were similar to those in the Pb-containing ores, emissions of ore smelting and Chinese coal, indicating the possible sources of Pb. Furthermore, the Pb isotope compositions in the O horizon were in agreement with those in aerosols and rainwater in southwest China. This further confirmed that Pb in the soils was probably from atmospheric deposition. However, based on the Pb isotope ratios, the China vehicle exhausts might be a minor factor affecting the Pb accumulation in the soils.

### Anthropogenic contribution to trace metals in the soils

Enrichment factor cannot indicate anthropogenic sources of trace metals in soil^24^; however, it shows contamination after a well-defined source. Based on the EFs (Fig. 3), phosphorus in the soils seemed to be influenced by anthropogenic impacts. Phosphorus is one of the main limiting nutrients for terrestrial ecosystems^25^, and is associated with complicated biogeochemical processes in mountain ecosystems, including the release from parent materials by weathering, mobilization via soil solutions, uptake by organisms, returning to forest floor by litterfall, and then adsorption by other minerals^26^. This made P different from trace metals that also presented notable enrichment in the O and A horizons. Copper and Zn, which are biologically active elements, showed significant correlation with P. They could be taken up directly from deeper soils by plants and then returned to forest floor by the detoxification, defoliation, mortality and decay of plants^19^,^27^. This mechanism could result in the marked element enrichments in topsoil. Therefore, we concluded that biogeochemical processes played a vital role in the enrichment of P, Cu and Zn in the upper layer of the soils.

As discussed above, Ag, Cd, Pb, Sb and even Zn in the O and A horizons suffered from anthropogenic impacts, and their EFs revealed a high contamination level (Fig. 3). Along the elevation gradient, the high enrichments of these metals in the O and A horizons were also observed in the high elevation area (Fig. 4), which was probably related to anthropogenic contribution via long-range atmospheric transport. To evidence this, a binary mixing model of Pb isotopes (Eq. 2) was used to quantitatively evaluate anthropogenic contribution to Pb. The percentage of anthropogenic Pb in the O and A horizons (especially in the O horizon) showed a marked change point at the elevation of 3614 m a.s.l., and the higher anthropogenic Pb existed in the low and high elevation zones, respectively (Fig. 6).

There were few local pollution sources in the area of Mt. Gongga^28^. Yang *et al*.^29^ analyzed the element composition of PM_2.5_ and PM_10_ at the elevation of 1600 m a.s.l. on the eastern slope of Mt. Gongga, and concluded that several trace metals (Ag, Cu, Pb and Zn) were sourced from long-distance atmospheric transport. Studies of aerosols on the eastern slope of Mt. Gongga^29^ and wet deposition in the central TP^30^, combined with the NOAA HYSPLIT model, have indicated that trace metals could reach the remote high-elevation area of the Tibetan Plateau through long-range atmospheric transport in the summer monsoon season. The South Asia regions (*e.g.,* India, Bangladesh and Pakistan) are still a primary source of pollutant emissions^31^,^32^, and anthropogenic metals from South Asia could likely be transported to Mt. Gongga via the summer monsoon. Furthermore, trace metals from mining, metal smelting and coal combustion in southwestern China represented another possible source^33^,^34^. For example, Tian *et al*.^35^ recently reported the anthropogenic atmospheric releases of various hazardous trace elements by China’s coal-fired power plants. The concentrations (mg/kg) of Cd, Pb and Sb in consumed coal in southwest China (*e.g.,* 0.80, 41.7 and 1.19 in Yunnan Province, respectively; 1.74, 29.6 and 1.76 in Sichuan Province, respectively; 1.19, 29.9 and 2.05 in Chongqing Municipality, respectively; and 0.79, 23.9 and 6.00 in Guizhou Province, respectively) were obviously higher than those in other parts of China.

### Factors dominating the trace metal distribution in the soils

Alpine regions exhibit substantial elevation-dependent differences in the climatic, topographical and biological characteristics, such as increased atmospheric deposition and cloud water interception due to low temperatures. Thus, these regions act as regional convergence areas for pollutants. Our data showed a distinct distribution of trace metals in the soils along the elevation gradient (Fig. 2). The trace metals in the soils had a high affinity for SOC, especially for Ag, Cd, Pb and Sb (Fig. 7); thus, their concentrations were normalized for SOC to isolate the dominant factors affecting the trace metal distribution in the soils (Fig. 8). Along the elevation gradient, two regions featured high concentrations of Cd, Pb, Sb and Zn (approximately 3000 and above 3700 m a.s.l., respectively). The concentrations of Cu were significantly higher at the lowest elevation, and the concentrations of Ag were high in the mid-elevation zone (approximately 3000 m a.s.l.). The distribution patterns of trace metals in the soils were not in agreement with the assumption that pollutants would increase with elevation in high mountains if there were no or rather weak local pollution^36^.

Many researchers have also observed the mid-elevation accumulation pattern of trace metals in mountain systems. Bacardit and Camarero^1^,^9^ attributed this distribution pattern in the snowpack and soils of the Central Pyrenees to local contamination. Zhang *et al*.^10^ indicated that the two types of rocks in the Mt. Everest region resulted in a varying distribution of trace metals in the soils. Yeo and Langley-Turnbaugh^37^, however, suggested that the middle elevations were the most ideal area for trace metal deposition on the Mt. Everest under the influence of wind. Gerdol and Bragazza^5^ concluded that cloud water was an important contributor to elevation-dependent trace metal distribution in alpine moss of the southeastern Alps. As discussed above, local contaminations were not a major source of trace metals in the soils. Therefore, we speculated that atmospheric deposition via the mountain cold condensation effect, especially precipitation, was one of the major factors controlling the elevation-dependent distribution of trace metals. For this purpose, we used the precipitation database compiled from three automatic meteorological stations (1600, 3000 and 4200 m a.s.l.)^38^ and the annual precipitation measured at another three elevations (2000, 2770 and 4000 m a.s.l.) in 2010 to estimate the precipitation pattern with elevation. A parabolic function of precipitation with elevation was obtained (y = −0.0005x^2^ + 2.9123x − 2402.2, r^2^ = 0.92), with which the relationship between element concentrations in the O horizon and precipitation was established (Fig. S3). An exponential increase in the concentrations of Ag, Pb and Sb with precipitation was observed, indicating the potential contribution of wet deposition. However, the biologically active elements (Cu and Zn) did not increase with precipitation. Cadmium, which was the most enriched toxic metal in the soils, was also not strongly related to precipitation. This indicated that, besides the wet deposition, the vegetation also played an important role in the metal distributions^39^.

The concentrations of Cu in the O and A horizons and Zn in the A horizon were relatively low between 2800 m and 3700 m a.s.l. (Figs 2 and 8), which was related to the uptake of plants. Coniferous trees have been found to accumulate more elements than broadleaf forests due to the duration of foliage^40^. The higher element concentrations in leaves and twigs of fir trees (*Abies fabri*) and rhododendron plants (*Rhododendron williamsianum*) have been observed at elevations between 3200 and 3400 m a.s.l. on the eastern slope of Mt. Gongga^41^. Meanwhile, Cu and Zn did not exhibit marked correlation with SOC (Fig. 7), and some other micronutrients negatively correlated with SOC (Fig. S4). This confirmed that plants re-distributed these elements in the soils through biogeochemical processes. Moreover, the lowest concentrations of trace metals were observed in the soils near the timberline (3500–3700 m a.s.l.) despite the normalization for SOC (Figs 2 and 8). This was not supported by the low biomass, productivity and tree growth in this zone (Fig. S5a,b). On the one hand, the low quantity of litterfall has been observed in this zone (Fig. S5c), which might directly reduce the quantities of trace metals returning to the soils. On the other hand, the precipitation is mainly in the form of snow in the timberline, and the trace metals deposited in the soils tend to run off after the snow melting. In addition, soil acidification also affects element accumulation in soil^42^. At the elevations of 2400–3800 m a.s.l., the soil pH was between 3.30 and 5.99 (Table 1), which was similar to the results reported by He *et al*.^43^. Coniferous trees have a greater capacity to acidify soil more than deciduous trees^44^, which accelerated elemental leaching and loss from the soils.

The canopy filtering effect is another mechanism that affects the distribution of trace metals in soil^17^,^45^,^46^. Forest filtering is mainly modulated by differences in the leaf area index (LAI, area of leaf/area of ground surface). A high LAI was observed in the mixed and coniferous forests (2900–3600 m a.s.l.) on the eastern slope of Mt. Gongga (Fig. S6). The high LAI suggested greater interception of Ag, Cd, Pb and Sb by plant uptake and adsorption onto the surfaces of leaves. These metals would return to the soils through litterfall. The significant positive correlation of these metals with SOC supported this assumption (Fig. 7). However, the forest canopy could also intercept significant quantities of precipitation and cloud water droplets^47^,^48^,^49^, which might reduce the deposition of trace metals on the forest floor. On the eastern slope of Mt. Gongga, the area of approximately 3000 a.s.l. received abundant precipitation (Table 1). This water would leach the various dry-deposited trace metals from tree surfaces, and carry them to soils.

### Conclusions

The remote high mountain in the eastern Tibetan Plateau suffered from contamination by anthropogenic metals. The effects of vegetation (*e.g.*, uptake, litterfall, canopy filtering) and wet deposition caused Ag, Cd, Pb and Sb accumulated in the soils of mid-elevation areas (approximately 3000 m a.s.l.). The mountain cold trapping effect increased trace metal (*e.g.,* Cd, Pb, Sb and Zn) deposition above the timberline through long-range atmospheric transport. This was an important finding for the understanding and further modeling of trace metal transport processes and mechanisms at regional and global scales.

## Materials and Methods

### Study area

**Figure 1:**
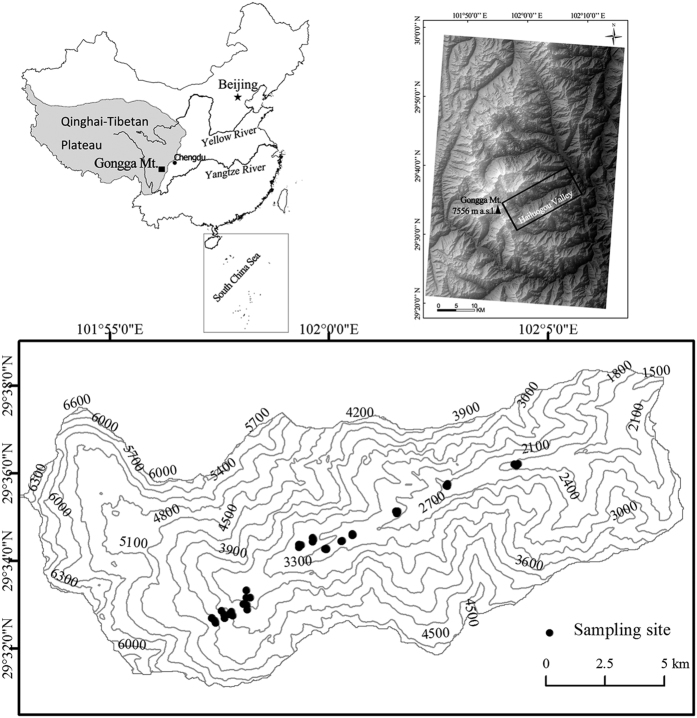
The study area and sampling sites on the eastern slope of Mt. Gongga. The map was created by the software ArcGIS 9.3 for Windows.

**Figure 2:**
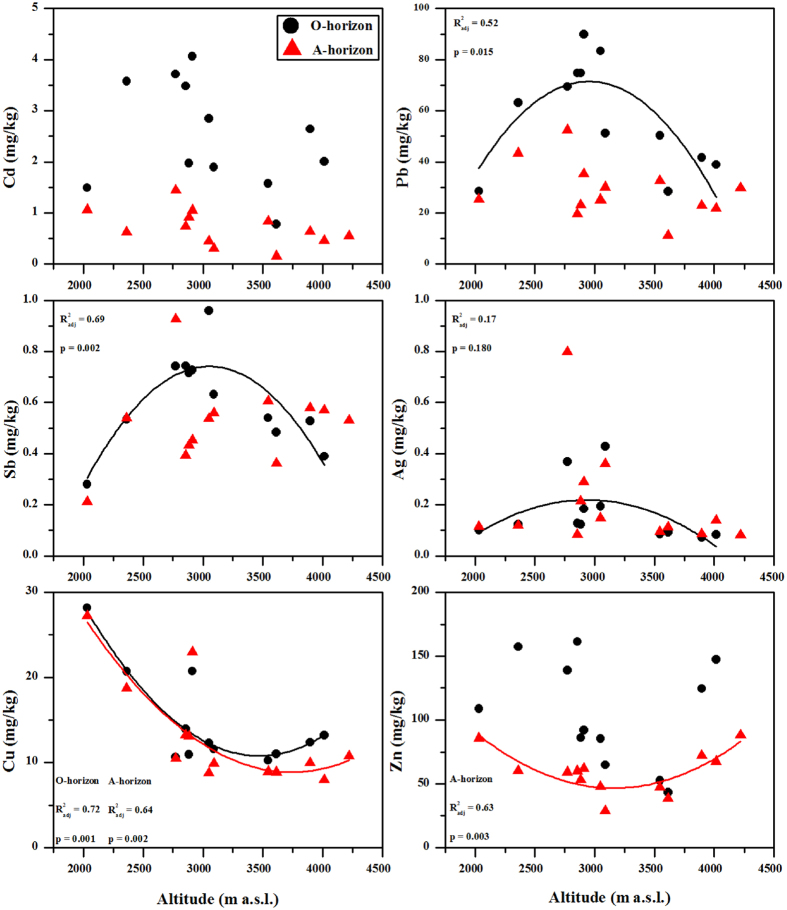
Elevation-dependent distribution of trace metals in the O and A horizons. The concentrations of trace metals were the medians from three repeated soil profiles.

**Figure 3:**
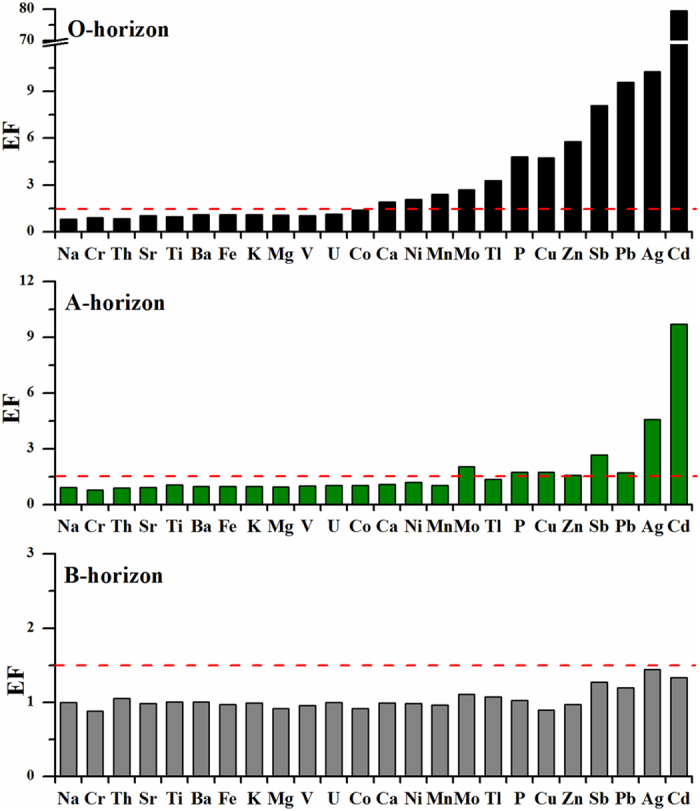
Enrichment factors (EFs) of elements (median values) in the soils.

**Figure 4:**
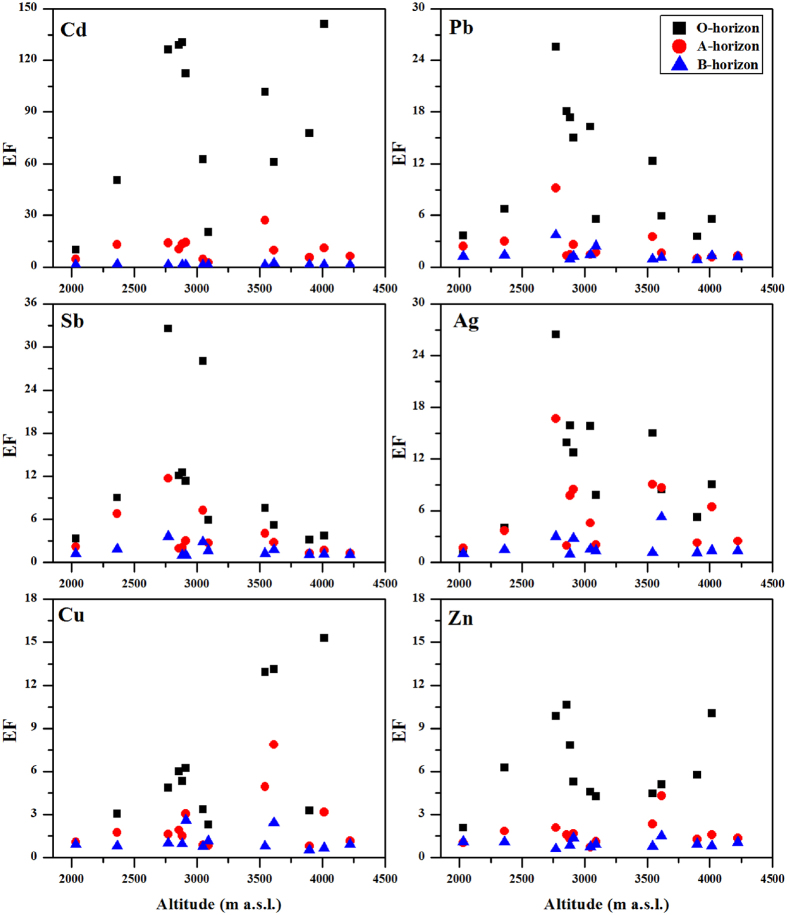
The EFs of trace metals in the soils along the elevation gradients. The values of EFs were the medians from three repeated soil profiles.

**Figure 5:**
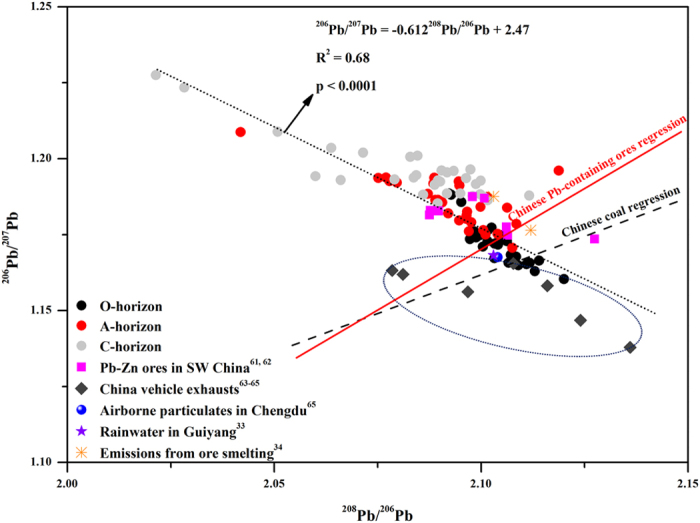
The diagram of Pb isotope compositions in the soils of the eastern slope of Mt. Gongga and other materials. The data of Chinese coal regression were cited from Refs 55, 56, 57, 58, and those of Chinese Pb-containing ores regression were from Refs 34,56,58,59,61, 62, 63, 64, 65.

**Figure 6:**
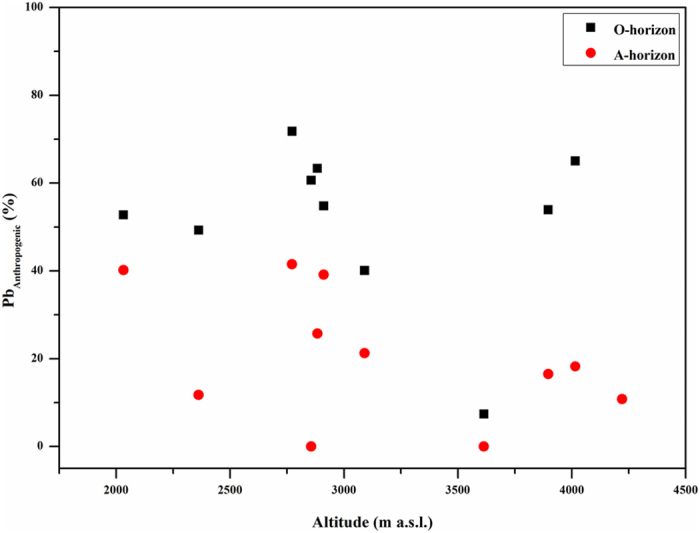
Percentage of anthropogenic Pb (mean values) in the O and A horizons along the elevation gradients.

**Figure 7:**
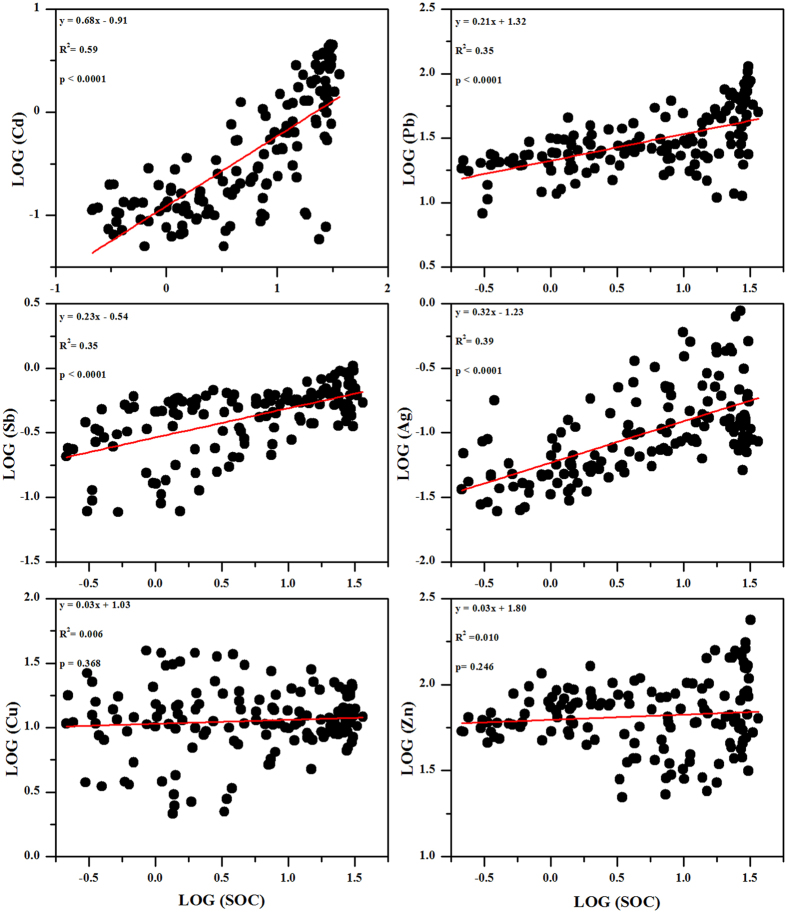
Relationships between trace metals and SOC in the soils (n = 153). The concentrations of trace metals and SOC were logarithmically transformed (LOG).

**Figure 8:**
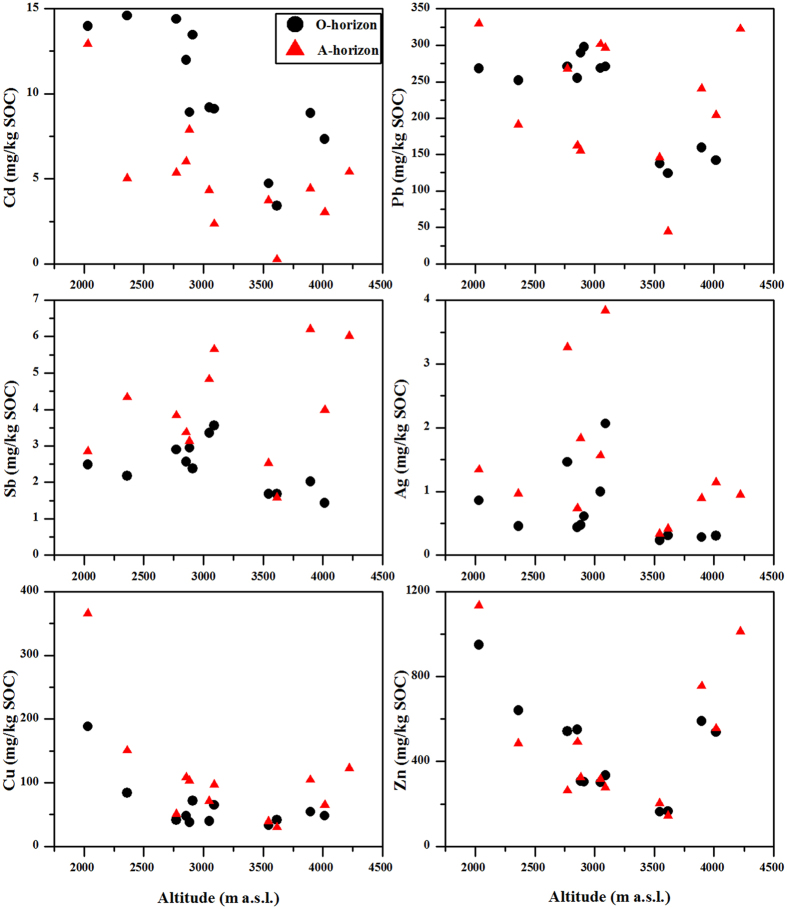
Concentrations of trace metals normalized for SOC in the soils along the elevation gradient. The concentrations of trace metals were the medians from three repeated soil profiles.

Soils were sampled on the eastern slope of Mt. Gongga within the Hailuogou Glacier valley area in the eastern Tibetan Plateau (Fig. 1). The climate on Mt. Gongga is typical temperate monsoon. The geomorphology is typified by high mountains and deep valleys, with large elevation differences (1100–7556 m a.s.l. within 26 km horizontal distance) and intense eroding cutting. The main parent material components are glacial debris and colluvial deposits derived from weathered Cenozoic feldspar granite and Permian quartz schist. Many debris flow gullies and deposits were formed by frequent debris flows along the elevation transect^50^. The characteristics of the vegetation distribution and soil types with elevation, as well as the meteorological information, are summarized in Table 1.

### Sample collection and analysis

After a preliminary survey of the topography and vegetation, the sampling sites were selected to include all vegetation zones (Table 1). Soil sampling along a large elevation gradient (2000–4500 m a.s.l.) was conducted in September 2010 at thirteen elevations: 2032, 2362, 2772, 2856, 2883, 2911, 3048, 3090, 3544, 3614, 3896, 4015 and 4221 m a.s.l. (Fig. 1, Table S1). Because of hard accessibility and the differences of vegetation distribution along the elevation gradient, the sampling sites were selected unevenly at different elevations. At each site, three soil profiles were hand-dug till the bedrock. Four soil horizons were divided according to the primary features of the soils (Table S1): the O horizon represents the organic soils with brown color and decomposition/semi-decomposition organic materials; the A horizon represents the mineral soils with dark brown color and humus; the B horizon represents the mineral soils with yellowish brown color and illuvial and/or eluvial materials; and the C horizon represents the soil parent materials. Due to the different soil development among the sampling sites, the B horizon at the site of 2856 m a.s.l. and the O horizon at the site of 4221 m a.s.l. were absent. In total, 153 samples (35 from O horizons, 41 from A horizons, 36 from B horizons, and 41 from C horizons) were collected.

Fresh soil samples were air-dried at room temperature for several weeks. Then, the soils were sieved to <2 mm to remove plant residues and coarser particles, and were pulverized by an agate mortar to pass through a 200-mesh Nylon screen. The concentration of soil organic carbon (SOC), pre-treated with 1 mol/L HCl to remove carbonates, was determined by a FlashEA1112 elemental analyzer linked to a Thermo Delta^Plus^ Advantage mass spectrometer. The standard reference material (GSS-11) was used during the measurement of SOC, and the standard deviations were <10% of the certified value.

The soil samples were digested with the nitric acid, hydrochloric acid, hydrofluoric acid and perchloric acid^51^. Major and trace elements (Ag, Al, Ba, Ca, Cd, Co, Cr, Cu, Fe, K, Mg, Mn, Mo, Na, Ni, P, Pb, Sb, Sr, Th, Ti, Tl, U, V and Zn) were analyzed using an American Leeman Labs profile inductively coupled plasma-atomic emission spectrometer (ICP-AES) and inductively coupled plasma-mass spectrograph (ICP-MS). Standard solution SPEX^TM^ from the US was used as the standard. Quality control was assured by the analysis of duplicate samples, blanks and reference materials (GSD-9 and GSD-11, Chinese geological reference materials). According to the analysis of the repeated samples and reference materials, the relative standard deviation (RSD) was less than 3% for ICP-AES and less than 5% for ICP-MS. The recovery of the reference materials was 92–108% for ICP-AES analysis and 90–110% for ICP-MS analysis.

Lead isotopes (^208^Pb, ^207^Pb and ^206^Pb) in the soils after the digestion described above were determined by ICP-MS (Agilent 7700x). International standard reference material (SRM981-NIST, US) was used for instrument calibration, and standard material (GBW04426, China) was for analytical control. The maximum deviations of both ^208^Pb/^206^Pb and ^207^Pb/^206^Pb ratios related to the repeated measurements of the GBW04426 Pb standard were less than 0.002, and the RSD of which were 0.09 and 0.18%, respectively.

### Calculations

The enrichment factors (EFs) of the elements in the soils were calculated as follows:


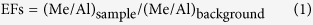


where (Me/Al)_sample_ represents the ratios of an element to Al in the sample, and (Me/Al)_background_ is the corresponding ratios in the background. The element concentrations in the UCC (upper continental crust) have often been selected as the background. However, the UCC was demonstrated not to reflect the local enrichment state of elements^52^. Therefore, the element concentrations in the C horizon were used as the background, and Al was selected as the reference element.

^206^Pb/^207^Pb ratios were applied to establish a binary mixing model to estimate the contribution of anthropogenic Pb to the soil. The function is as follow:


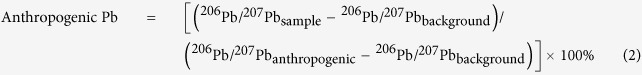


where ^206^Pb/^207^Pb_sample_ was the ratio of a given sample, and a value of 1.15 was used as the ^206^Pb/^207^Pb_anthropogenic_ based on the ratios in the O and A horizons and previous studies^53^,^54^. The ratios in the C horizon were selected as the background.

### Statistical analysis

One-Way ANOVA (Fisher Test, p < 0.05) was used to identify the significant differences in the mean values for soil variables at different horizons or elevations. Regression analysis and factor analysis were applied to establish the relationship between elements and other parameters in the samples. All statistical analysis in this study was performed by the software package SPSS 19.0 and Origin 8.0 for Windows.

## Additional Information

**How to cite this article**: Bing, H.J. *et al*. Vegetation and Cold Trapping Modulating Elevation-dependent Distribution of Trace Metals in Soils of a High Mountain in Eastern Tibetan Plateau. *Sci. Rep.* **6**, 24081; doi: 10.1038/srep24081 (2016).

## Supplementary Material

Supplementary Information

## Figures and Tables

**Table 1:** Geographic characteristics and vegetation distribution along the elevation gradient in the study area.

Vegetation zone	Elevation (m a.s.l.)	Soil type	Soil pH^a^	Dominant plants	Annual precipitation (mm)^b^	Annual temperature (°C)^b^
Broadleaf forest	<2400	Yellow-brown soil	6.49 (6.20–6.95)	*Lithocarpus cleistocarpus (Seem.) Rehd. Et Wils., Betula insignis*	1020–1600	7.1–12.8
Broadleaf-coniferous forest	2400–2900	Brown soil	4.66 (3.40–5.32)	*Picea brachytyla, Betula insignis*	1600–1870	4.5–7.1
Coniferous forest	2900–3800	Dark-brown soil	4.40 (3.30–5.99)	*Abies fabri*	1870–1947	0.9–4.5
Shrub	3800–4000	Meadow soil	4.62 (3.88–6.13)	*Rhododendron cephalanthum, R. Phaeochrysum*	—	—
Meadow	4000–4600	Meadow soil	4.94 (4.70–5.27)	*Kobresia, Potentilla, Festucaovina*	1040	−5.7—0.9

^a^Data were detected *in situ*. Values in the brackets were the ranges of pH, which represented the values of surface soil (0–15 cm).

^b^Data from Wu *et al*.^38^ and Gao and Peng^60^.

**Table 2:** Concentrations of elements in the soils on the eastern slope of Mt. Gongga.

Elements	UCC^a^	Chinese UCC^b^	Chinese sedimentary layer^b^	O-horizon (n = 35)		A-horizon (n = 41)		B-horizon (n = 36)		C-horizon (n = 41)	
				Median	MAD	Median	MAD	Median	MAD	Median	MAD
Ag	0.05	0.054	0.051	0.13	0.04	0.12	0.04	0.07	0.04	0.05	0.01
Al	80.4	75.1	50.8	16.7	4.2	47.3	8.8	66.9	3.7	69.8	2.8
Ba	550	660	260	234	85	455	118	738	309	956	281
Ca	30	32.5	74.5	15.5	5.9	24.5	8.4	29.7	4.9	32.1	6.5
Cd	0.098	0.049	0.053	2.33	1.03	0.63	0.32	0.14	0.05	0.12	0.03
Co	10	24	33	4.3	1.4	9.4	4.5	11.5	3.1	13.3	2.0
Cr	35	25	52	82.9	19.7	174	78.3	295	149	384	155
Cu	25	32	28	12.4	2.1	10.8	2.5	10.5	4.1	12.0	3.8
Fe	35	27.6	33.1	11.0	2.7	25.0	7.6	35.3	8.3	40.0	6.3
K	28	25.7	16.6	6.3	1.8	14.7	3.4	19.7	5.0	22.4	4.0
Mg	13.3	7.86	13.7	5.3	2.0	10.7	4.4	15.6	4.4	17.6	3.2
Mn	600	500	260	398	194	466	230	594	160	646	109
Mo	1.5	1.6	0.56	0.61	0.12	0.86	0.18	0.81	0.14	0.79	0.17
Na	28.9	22.9	22.5	3.3	1.1	10.9	3.1	17.0	2.5	17.6	2.1
Ni	20	13	25	12.4	3.6	21.0	7.1	23.4	7.3	26.2	5.1
P	700	750	690	1190	153	1070	149	930	248	900	395
Pb	20	19	11	56.3	16.4	25.3	5.7	24.2	6.1	22.1	3.7
Sb	0.2	0.19	0.39	0.61	0.13	0.53	0.09	0.48	0.14	0.32	0.16
Sr	350	470	330	106	32	238	95	341	198	492	180
Th	10.7	25	8.7	3.6	0.8	9.1	4.3	12.4	4.8	15.1	8.6
Ti	3000	2530	2650	1260	317	3310	943	3500	778	4840	766
Tl	0.75	0.88	0.50	0.35	0.07	0.40	0.06	0.45	0.07	0.46	0.09
U	2.8	8.1	2.0	1.1	0.2	2.2	0.8	3.0	1.0	3.6	1.0
V	60	63	54	30.7	8.2	81.0	20.9	111	25.7	114	14.4
Zn	71	51	45	94.9	39.5	60.3	13.8	58.1	18.3	64.6	13.0

The element concentrations from the UCC, Chinese UCC and Chinese sedimentary layer were also shown.

Note: The units of Al, Na, Fe, K, Mg and Ca were mg/g, otherwise mg/kg;

MAD = Median |Yi−Y|, where Y is the median of the data and |Y| represents the absolute value of Y.

^a^UCC = upper continental crust. Cited from Taylor and McLennan^15^.

^b^Cited from Li^16^.

**Table 3:** Factor loadings of variables by factor analysis (Extraction method: Principal component analysis; Rotated with Varimax).

Components				
	1	2	3	4
Al	**0.946**	−0.068	−0.073	0.112
Ba	**0.746**	**−0.498**	0.313	−0.124
Ca	**0.668**	0.294	0.054	−0.341
Cr	**0.734**	−0.108	−0.159	−0.320
Fe	**0.864**	0.266	0.065	0.284
K	**0.840**	−0.314	0.170	0.072
Mg	**0.785**	0.330	0.053	−0.099
Mn	**0.529**	**0.411**	**0.435**	0.009
Na	**0.811**	−0.010	−0.234	−0.226
Sr	**0.745**	**−0.446**	0.281	−0.292
Ti	**0.811**	0.092	−0.012	0.398
V	**0.879**	0.156	0.066	0.318
Co	**0.824**	**0.485**	0.113	0.012
Ni	**0.684**	**0.566**	−0.154	−0.040
Th	**0.603**	**−0.554**	0.214	−0.149
U	**0.683**	**−0.498**	0.208	0.005
Mo	0.204	−0.052	0.083	**0.625**
Tl	0.306	−0.149	**0.547**	0.386
P	−0.037	0.043	**0.658**	−0.149
Cu	0.143	**0.903**	0.026	0.123
Zn	−0.116	**0.590**	0.592	−0.300
Ag	**−0.472**	−0.016	0.088	**0.561**
Cd	**−0.707**	0.360	**0.425**	−0.235
Pb	**−0.624**	0.132	**0.533**	0.036
Sb	**−0.561**	−0.390	**0.541**	0.112
Eigenvalue	11.0	3.63	2.48	1.81
Variance (%)	30.0	25.9	11.6	8.2
Cumulative variance (%)	30.0	55.9	67.5	75.7
