# Supplementary material for: Vegetation and Cold Trapping Modulating Elevation-dependent Distribution of Trace Metals in Soils of a High Mountain in Eastern Tibetan Plateau
Source: Sci Rep. 2016 Apr 7;6:24081. doi: 10.1038/srep24081 (PMC4823730; doi:10.1038/srep24081)
Supplement: Supplementary Information [file srep24081-s1.pdf]

Supplementary Information for:

Submitted to: **Scientific Reports**

Title: Vegetation and Cold Trapping Modulating Elevation-dependent Distribution of Trace Metals in Soils of a High Mountain in Eastern Tibetan Plateau

Authors: Haijian Bing, Yanhong Wu [*], Jun Zhou, Rui Li, Ji Luo, Dong Yu

Institute affiliation: Alpine Ecosystem Observation and Experiment Station of Gongga Mountain, The Key Laboratory of Mountain Surface Processes and Ecological Regulation, Institute of Mountain Hazards and Environment, Chinese Academy of Sciences, Chengdu 610041, China

Number of pages: 10

Number of tables: 2

Number of figures: 6

Number of references: 4

[*] Corresponding author: Prof. Yanhong Wu (PhD), E-mail: yhwu@imde.ac.cn, Tel: 86 28 85257118, Fax: 86 28 85257118, Postal address: Institute of Mountain Hazards and Environment, Chinese Academy of Sciences, #9, Block 4, Renminnanlu Road, Chengdu, China

Table S1 Description of the sampling sites and soil depth on the eastern slope of Mt. Gongga

Site	Latitude (N)	Longitude (E)	Altitude (m a.s.l.)	Soil depth (cm) [a]			
				O-horizon	A-horizon	B-horizon	C-horizon
1	29°36′12.0″	102°4′14.1″	2032	0~3	4~15	16~35	>40
2	29°35′43.1″	102°2′40.9″	2362	0~3	4~15	16~22	>38
3	29°35′7.9″	102°1′32.0″	2772	0~7	8~15	16~30	>60
4	29°34′36.0″	102°00′32.1″	2856	0~3	4~13	--	>61
5	29°34′28.9″	102°00′19.4″	2883	0~5	6~9	10~12	>50
6	29°34′21.2″	102°00′3.5″	2911	0~4	5~8	9~13	>48
7	29°34′31.6″	101°59′37.6″	3048	0~3	4~7	8~12	>35
8	29°34′21.8″	101°59′19.1″	3090	0~3	4~9	10~22	>30
9	29°33′9.3″	101°58′7.0″	3544	0~2	3~5	6~9	>30
10	29°32′59.7″	101°58′7.3″	3614	0~2	3~7	8~15	>25
11	29°32′46.7″	101°57′43.6″	3896	0~2	3~10	10~17	>25
12	29°32′46.3″	101°57′36.0″	4015	0~3	4~11	12~24	>30
13	29°32′37.8″	101°57′23.3″	4221	--	0~4	5~7	>20

[a] The mean depth of soil horizons presented here was measured in situ from three profiles at each site.

Table S2 Concentrations of soil organic carbon (SOC, %) on the eastern slope of Mt. Gongga

	Soil horizon			
	O	A	B	C
Range	10.7-36.6	4.3-28.2	0.5-18.5	0.2-7.3
Mean	25.6^{a}	14.4^{b}	4.5^{c}	1.4^{d}
Median	27.8	12.3	2.7	1.0
SD	6.2	7.5	4.6	1.4

The different lowercases represent the significant difference of SOC in the soil ($p < 0.05$, Fisher Test)

Figure S1 Concentrations of soil organic carbon (SOC, %) in the different vegetation zones of the eastern slope of Mt. Gongga. The different lowercases in the top of the histograms represent the significant difference of SOC among the vegetation zones ($p < 0.05$, Fisher Test). The error bars represent the standard errors of the data

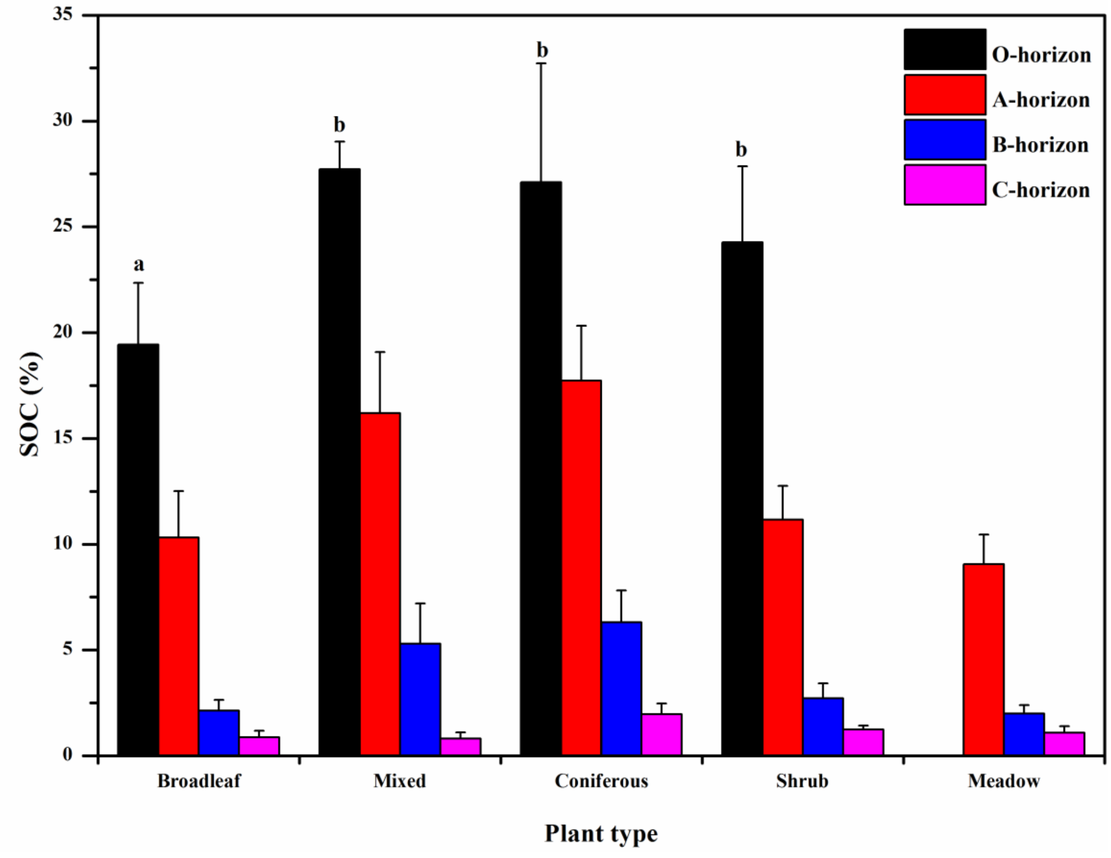

Figure S2 Component scores of variables by factor analysis

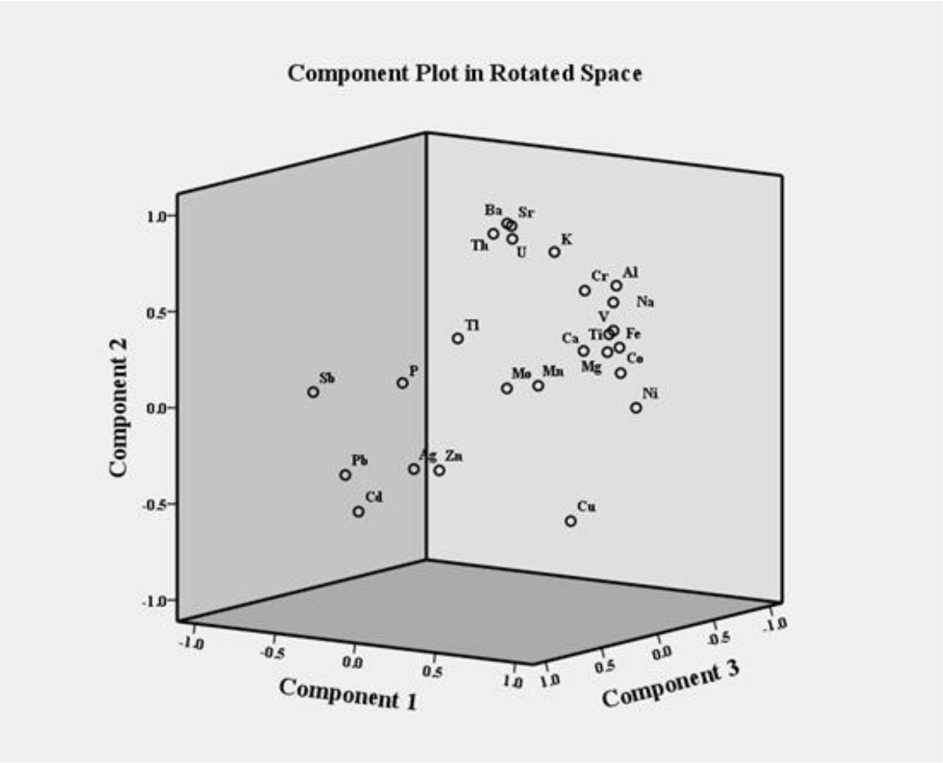

Figure S3 Relationships between trace metal concentrations in the O horizon and precipitation

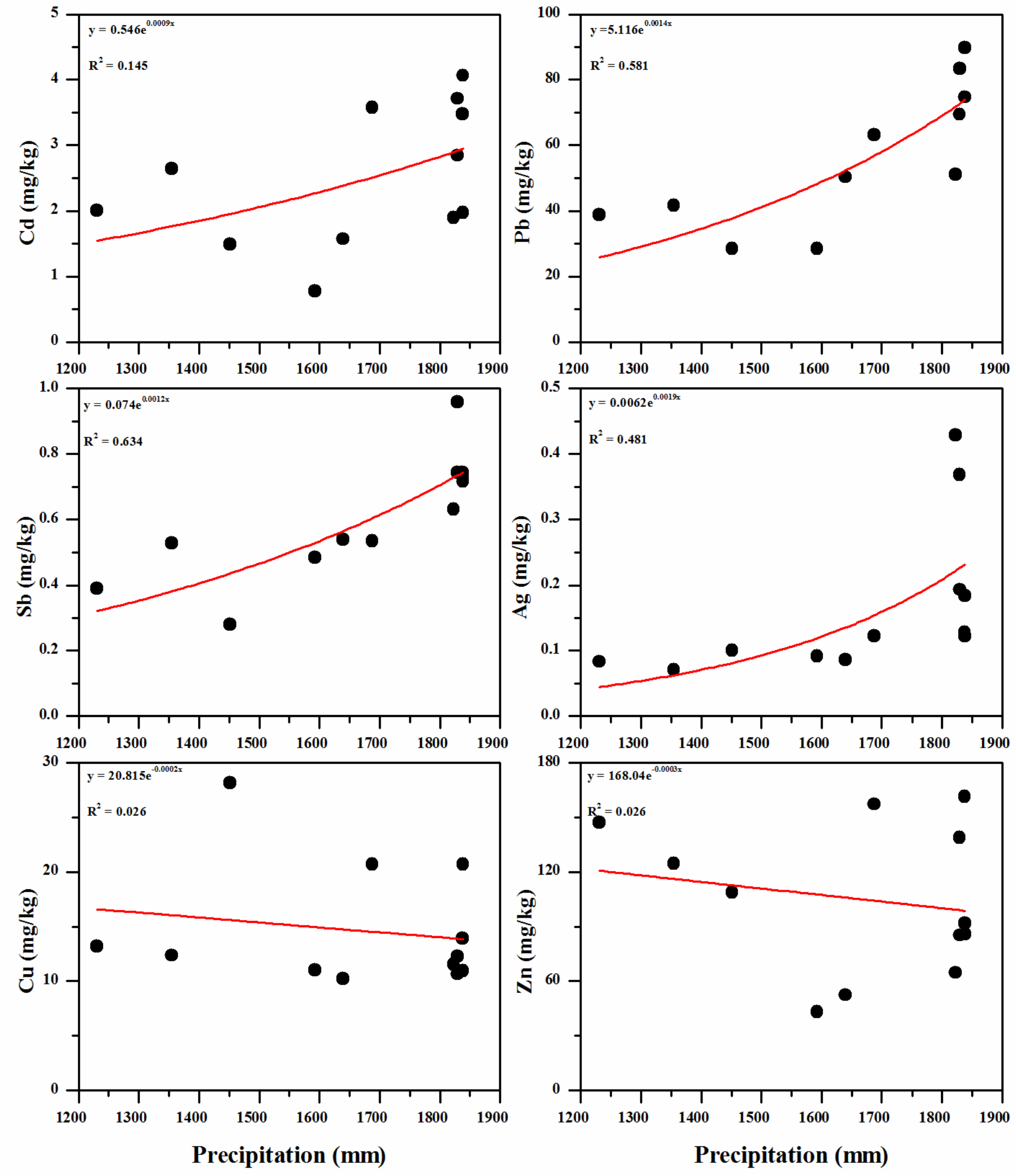

Figure S4 Correlations of Cr, Co, Ni, Tl, Fe and Mn with SOC in the soils (n = 153)

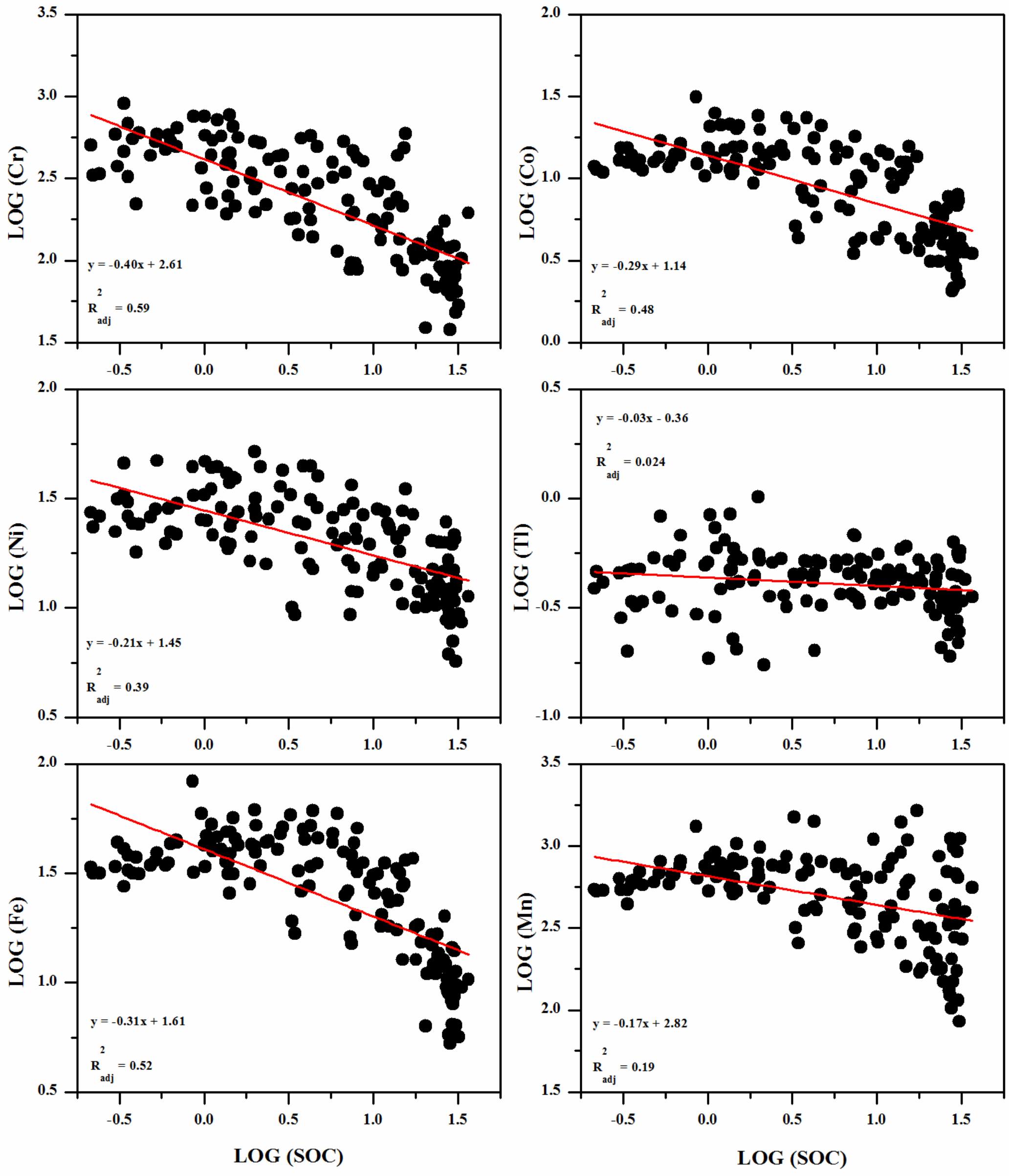

Figure S5 The biomass and productivity (a), tree height, basal diameter and volume (b), and litterfall (c) in the forests on the eastern slope of Mt. Gongga. The data of biomass, productivity, tree height, basal diameter and volume were cited from Luo et al. (2000a, b), and those of litterfall were cited from Luo et al. (2003)

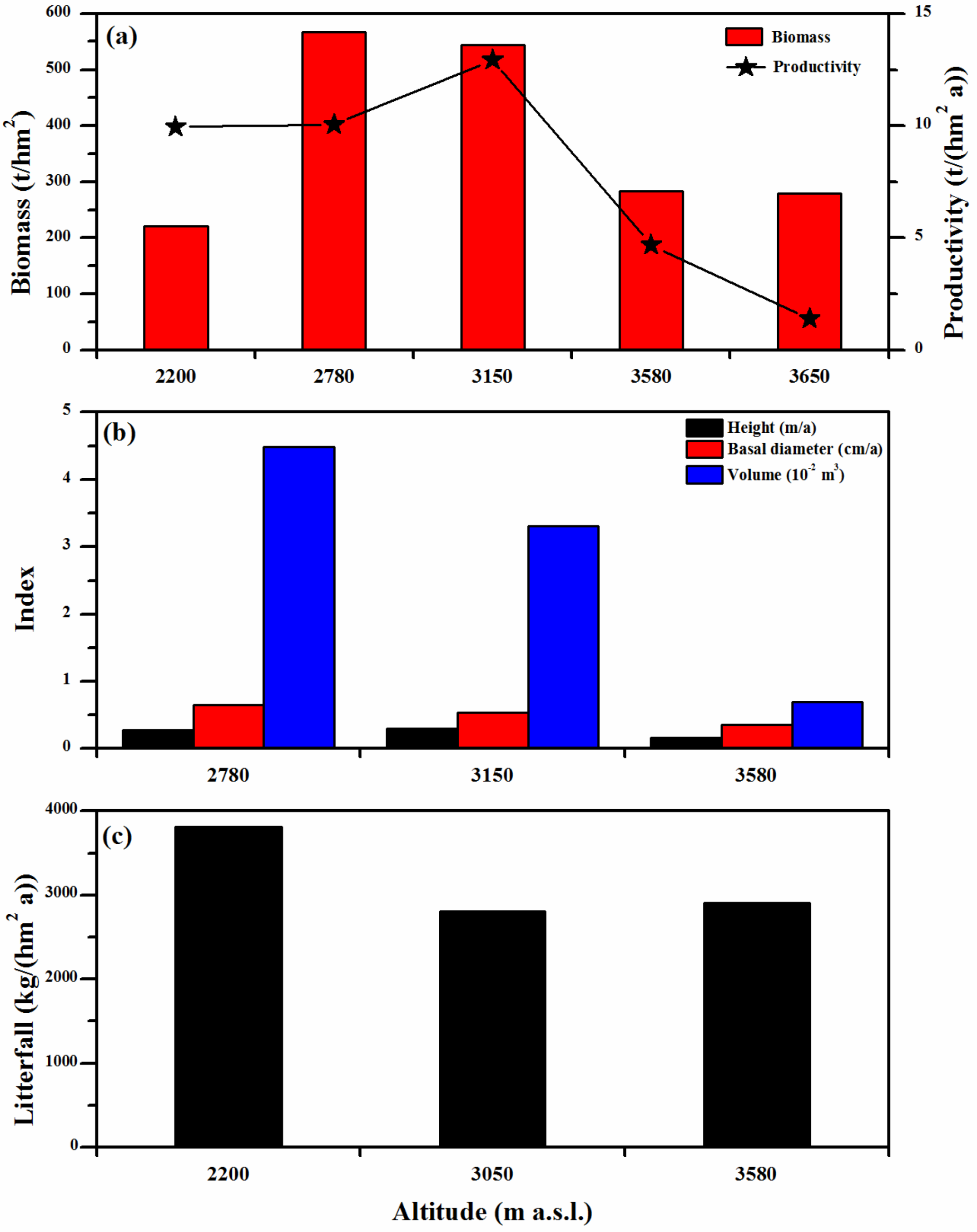

Figure S6 Leaf area index (LAI) in the forests on the eastern slope of Mt. Gongga. The error bars represent the standard deviation of the data from Luo et al. (2004)

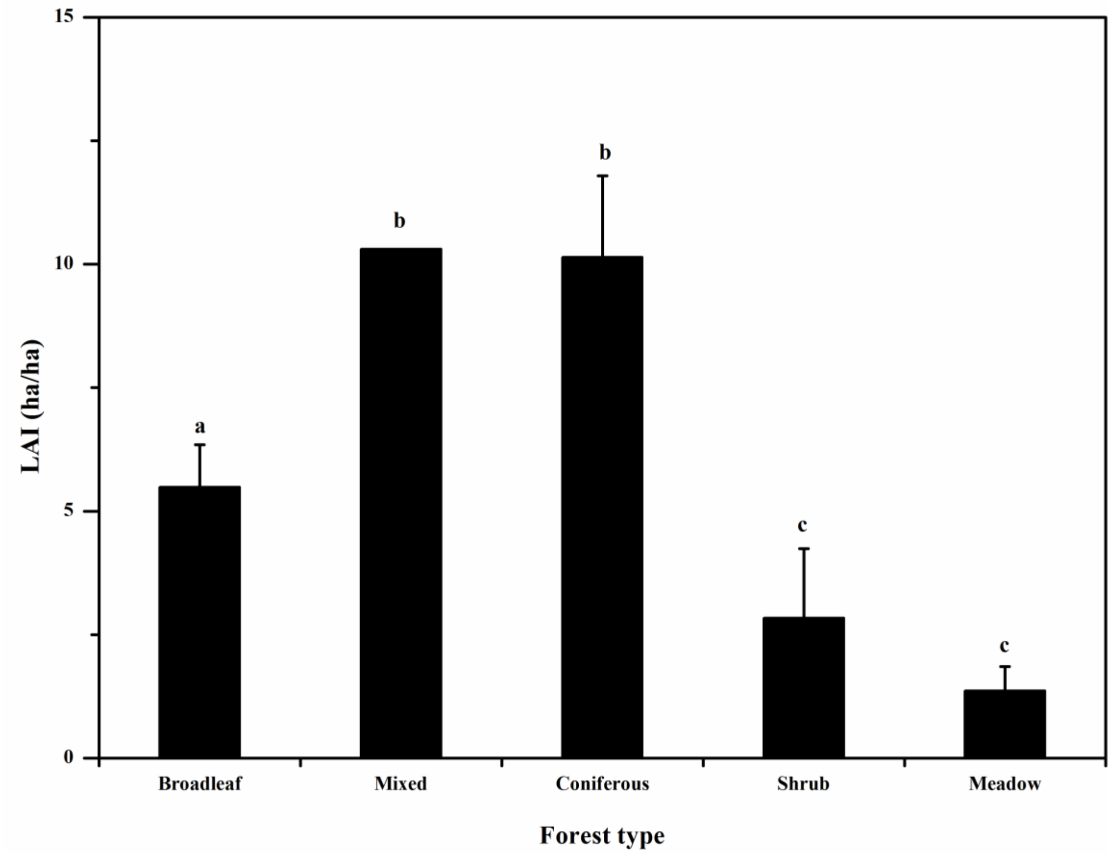

References

Luo, J., Yang, Z. & Yang, Q.W. A study on the biomass and productivity of forest on the Gongga Mountain. Acta Phytoecologica Sinica, **24**, 191-196 (2000a). (In Chinese with English abstract)

Luo, J., Cheng, G.W., Yang, Z. & Yang, Q. W. Growth dynamics of dominant tree species in dark coniferous forests on Gongga Mountain. Acta Phytoecologica Sinica, **24**, 22-26 (2000b). (In Chinese with English abstract)

Luo, J., Cheng, G.W., Chen, B.R. & Li, W. Characteristic of forests litterfall along vertical spectrum on the Gongga Mountain. Journal of Mountain Science, **21**, 287-292 (2003). (In Chinese with English abstract)

Luo, T.X., Pan, Y.D., Ouyang, H., Shi, P.L., Luo, J., Yu, Z.L. & Lu, Q. Leaf area index and net primary productivity along subtropical to alpine gradients in the Tibetan Plateau. Global Ecology and Biogeography, **13**, 345-358 (2004).
